# Amphiphilic Polypeptides for VEGF siRNA Delivery into Retinal Epithelial Cells

**DOI:** 10.3390/pharmaceutics12010039

**Published:** 2020-01-02

**Authors:** Olga Osipova, Vladimir Sharoyko, Natalia Zashikhina, Natalya Zakharova, Tatiana Tennikova, Arto Urtti, Evgenia Korzhikova-Vlakh

**Affiliations:** 1 Institute of Chemistry, Saint Petersburg State University, Universitetskii pr. 26, 198584 Saint Petersburg, Russia; olya_osipova_06_01@mail.ru (O.O.); sharoyko@gmail.com (V.S.); nzashihina@bk.ru (N.Z.); tennikova@mail.ru (T.T.); arto.urtti@uef.fi (A.U.); 2 Institute of Macromolecular Compounds, Russian Academy of Sciences, Bolshoy pr. 31, 199004 Saint-Petersburg, Russia; na_zar@inbox.ru; 3 School of Pharmacy, University of Eastern Finland, Yliopistonranta 1 C, 70210 Kuopio, Finland; 4 Faculty of Pharmacy, University of Helsinki, Viikinkaari 5 E, 00790 Helsinki, Finland

**Keywords:** amphiphilic polypeptides, self-assembly, nanoparticles, siRNA delivery, VEGF, gene silencing

## Abstract

Polyethyleneimine, poly-L-lysine, chitosan and some others cationic polymers have been thoroughly studied as nucleic acid delivery systems in gene therapy. However, the drug release from these systems proceeds at a very low rate due to extremely high binding between a carrier and gene material. To reduce these interactions and to enhance drug release, we developed a set of amphiphilic polypeptides containing positively and negatively charged amino acids as well as a hydrophobic one. The copolymers obtained were characterized by size-exclusion chromatography, static light scattering, HPLC amino acid analysis and ^1^HNMR spectroscopy. All copolymers formed particles due to a self-assembly in aqueous media. Depending on polypeptide composition, the formation of particles with hydrodynamic diameters from 180 to 900 nm was observed. Stability of polymer particles, loading and release efficiency were carefully studied. Cellular uptake of the particles was efficient and their cytotoxicity was negligible. The application of polymer carriers, containing siRNA, to vascular endothelial growth factor (VEGF-A165) silencing of ARPE-19 cells was successful. The gene silencing was confirmed by suppression of both messenger RNA and protein expression.

## 1. Introduction

Diabetic retinopathy (DR) and age-related macular degeneration (AMD) are among the most common ocular diseases that lead to the impairment or loss of vision [1,2]. Proliferative neovascular forms of DR and AMD are characterized by the formation of new vessels from the existing ones, because the proliferation and migration of endothelial cells are stimulated by over-expression of vascular endothelial growth factor (VEGF) [3]. Inhibition of VEGF via intraocular injections of anti-VEGF drugs are the corner-stone approach in the treatment of AMD and diabetic macular edema that is associated with DR. Currently, a common clinical therapy is based on application of the monoclonal antibodies (mABs) that specifically bind to VEGF, and neutralize its function. Such recombinant monoclonal antibodies as bevacizumab and its Fab-fragment ranibizumab bind with high affinity to the site present in all VEGF isoforms [4,5]. Besides antibodies, there is also the aptamer pegaptanib (28-nucleotide RNA aptamer) that was registered for the treatment of neovascular AMD fifteen years ago [4,5]. In spite of the existing clinical therapeutics, some drawbacks still exist in the treatment of neovascular AMD. Monthly or bimonthly injections into the vitreous of the eye constitute a major burden to the patients and healthcare. Furthermore, the intravitreal injections may lead to some complications, such as retinal detachment, intraocular pressure increase, ocular infection, and hyperemia.

In contrast to the mABs and aptamers, small-interfering RNAs (siRNAs) do not block protein function but play a key role in the post-transcriptional gene silencing [6]. siRNAs have a great potential as drugs, including the treatment of ocular diseases. For instance, phase II clinical trials of ocular siRNA treatments were reported [7]. These trials by the Quark and Pfizer companies aim at developing treatments for wet AMD, glaucoma and some other ocular diseases.

To inhibit intracellular VEGF synthesis, usually picomole quantities of siRNA are necessary [8]. Moreover, siRNAs provide a long therapeutic effect with a single administration and thereby may reduce the risks and burden associated with intravitreal anti-VEGF therapy. Another benefit of siRNA is the simple synthesis and possibility to up-scale the production. However, the successful application of siRNAs is limited by the necessity to overcome several biological barriers [9]. The first one is the penetration across the cell membrane. The physicochemical properties of siRNAs, such as hydrophilicity, relatively large size and net negative charge, complicate their entrance to the cell. After cellular internalization, siRNA must escape the endosomes and reach the cytosol, where it must be involved into formation of the RNA-inducing silencing protein complex (RISC) and recognize the target mRNA. Furthermore, siRNA is subject to enzymatic degradation, but its stability can be improved with chemical modifications (e.g., phosphorothioate) and formulation techniques.

To overcome the obstacles, different siRNA delivery systems have been investigated. The carriers for siRNA delivery should meet some requirements. They must be biocompatible, biodegradable and they must have suitable combination of chemical-physical properties, including charge, size, stability, and possibility to surface functionalization. Currently, the encapsulation of the siRNAs into nanoparticles is based on lipids [10,11] or polymers [12,13], or their complexation with cell-penetrating peptides [14,15] or cationic-polymers [16,17,18,19]. Such technologies have been summarized in some recent reviews [20,21]. Self-assembled into micelles or liposomes, the cationic lipids can entrap siRNA and facilitate its cellular entry by endocytosis. However, a poor stability of such systems has induced a wave of studies on their covalent and non-covalent modification with PEG or its copolymers [22,23]. The preparation of complex delivery systems, for example, consisting of siRNA-cationic lipid complex encapsulated into folate-PEG-co-(polylactic acid-co-polyglycolic acid)-polyketal nanoparticles [12] or based on poly(ε-caprolactone)-co-PEG-co-poly(l-histidine) [13] were recently reported.

Protonable cationic polymers are among the leading candidates for nucleic acid binding and intracellular delivery. They are characterized with high buffering capacity at endosomal pH range (about 5.5–7.4) and it is hypothesized that they can mediate endosomal escape by acting as ‘proton sponges’. Polyethyleneimine (PEI), poly-L-lysine, chitosan and its derivatives are the most widely studied cationic polymers for nucleic acid delivery [17,19,24]. Despite high binding and cellular transfection efficiency of many polymers, they are not biodegradable (e.g., PEI, chitosan) or form stable polyplexes with limited release of nucleic acids (e.g., poly-L-lysine). To facilitate the release of nucleic acid from polyplex, recently, Pilipenko et al. suggested the introduction of heparin as concurrent polyanion to chitosan [25]. Chitosan-heparin polyplexes with different ratios of positive and negative polysaccharides were prepared and their efficiency for delivery of DNA and siRNA into human keratinocytes was estimated.

Following that idea, in present work, we developed a set of random amphiphilic polypeptides consisting of positively and negatively charged amino acids as well as a hydrophobic one. L-Lysine and L-glutamic acid were selected as oppositely charged amino acids to control siRNA binding and release. L-Phenylalanine or L-isoleucine provided polypeptide self-assembly and possibly facilitated delivery of siRNA across the cell membrane. The ratio between the three amino acids was varied to find the composition of polypeptides that provides optimal particle size, zeta potential, encapsulation efficiency, cytotoxicity and VEGF-A165 silencing in retinal pigment epithelial cells.

## 2. Materials and Methods

### 2.1. Materials

ε-Z-L-Lysine, γ-benzyl-L-glutamate, L-phenylalanine, L-isoleucine, triphosgene, α-pinene, trifluoroacetic acid (TFA), trifluoromethanesulfonic acid (TFMSA), and other reagents for NCA and polymer synthesis were ordered in Sigma-Aldrich (Darmstadt, Germany). All organic solvents, i.e., *N*,*N*-dimethylformamide (DMF), dimethyl sulfoxide (DMSO) 1,4-dioxane, petroleum ether, ethyl acetate, and some others were purchased from Vecton Ltd. (St. Petersburg, Russia), and purified before use. The buffer solutions were prepared by dissolving salts of ACS reagent grade purchased from Vecton Ltd. (St. Petersburg, Russia) in deionized water and afterwards the solutions were filtered through a 0.45-µm membrane Milex, Millipore Merck (Darmstadt, Germany). For purification of synthesized polymers, Spectra/Pore^®^ dialysis bags (MWCO: 1000, Rancho Dominguez, CA, USA) were used. Filter tubes (30,000) were ordered in Merck and in Sartorius (Göttingen, Germany).

SEC column calibration was performed with the use of poly(methyl methacrylate) (PMMA) standards (*M_w_* = 17,000–250,000; *Đ* ≤ 1.14) purchased from Supelco (Bellefonte, PA, USA). Poly-L-lysine (*M_w_* = 15,000–30,000) was a product of Sigma-Aldrich.

Model non-labeled and Cy3-labeled 23-base pairs duplex of oligothymidine and oligoadenine (oligo-dT-dA) were purchased from Biobeagle^TM^ (St. Petersburg, Russia). Dulbecco’s modified Eagle’s medium (DMEM), penicillin, streptomycin and fetal bovine serum (FBS) were obtained from BioloT (St. Petersburg, Russia).

The 27-base pairs (bp) double stranded RNA were selected as target to VEGF-A165 gene. siRNA sequence was the following: Sense—5′-CUUCCUACAGCACAACAAAUGUGAAUG-3′, antisense: 3′-GAAGGAUGUCGUGUUGUUUACACUUAC-5′. The same siRNA labelled with Cy5 was utilized for the visualization experiments. Cy5-labeled and non-labeled 27-bp VEGF siRNAs and scrambled 27-bp RNA for control (siControl) (sense 5′-GUAAGUGUAAACAACACGACAUCCUUC-3′, antisense: 3′-CAUUCACAUUUGUUGUGCUGUAGGAAG-5′ [21] were purchased from GenTerra (Moscow, Russia). The primers used for the target mRNA: VEGF forward and reverse primers, GAPDH forward and reverse primers were obtained from GenTerra.

Human retinal pigment epithelial (ARPE-19) cell line was a product of American Type Culture Collection (ATCC, Manassas, VA, USA), while HEK-293 (human embryonic kidney) and BEAS-2B (human bronchial epithelium) cell lines were obtained from the German Collection of Microorganisms and Cell Culture (DSMZ, Braunschweig, Germany).

### 2.2. Methods

#### 2.2.1. Synthesis and Characterization of Polypeptides

Polypeptides were synthesized by ring-opening polymerization (ROP) of α-amino acid *N*-carboxyanhydrides (NCA) as random structures. NCA monomers of Lys(Z), Glu(OBzl), Phe and Ile were prepared as described elsewhere [26]. In all cases, anhydrous dioxane was used as a solvent. Acquired NCAs were purified by recrystallization twice from anhydrous ethyl acetate/*n*-hexane. Yields: Lys(Z) NCA—87%, Glu(OBzl) NCA—89%, Phe NCA—74%, Ile NCA—69%.

The structure and purity of NCAs were testified by ^1^H-NMR at 25 °C in CDCl_3_. The spectra were recorded using a 400 MHz Avance instrument (Bruker, Karlsruhe, Germany). Lys(Z) NCA: δ 7.43–7.28 (m, 5H), 6.97 (s, 1H), 5.12 (s, 2H), 4.97 (s, 1H), 4.32–4.23 (t, *J* = 5.2, 1H) (s, 1H), 3.29–3.14 (m, 2H), 2.03–1.90 (m, 1H), 1.90–1.75 (m, 1H), 1.73–1.28 (m, 4H); Glu(OBzl) NCA: 2.05–2.39 (m, 2H), 2.63 (t, 2H), 4.39 (t, 1H), 5.17 (s, 2H), 6.40 (br. s., 1H), 7.39 (m, 5H); Phe NCA: 2.94–3.35 (m, 2H), 4.55 (m, 1H), 6.12 (s, 1H), 7.19–7.41 (m, 5H); Ile NCA: 0.836 (t, 3H), 0.871 (d, 3H), 1.236 (dq, 2H), 1.941 (qtd, 1H), 4.28 (d, 1H).

Two series of polypeptides, e.g., P(Lys(Z)-*co*-Glu(OBzl)-*co*-Phe) and P(Lys(Z)-*co*-Glu(OBzl)-*co*-Ile), were synthesized using *n*-hexylamine as initiator. The NCA/initiator molar ratio was 100. The following initial ratio of NKAs were used for the synthesis: (1) Lys(Z)/Glu(OBzl)/Phe = 60/20/20 (KEF1), 40/40/20 (KEF2) and 20/60/20 (KEF3); (2) Lys(Z)/Glu(OBzl)/Ile = 60/10/30 (KEI1), 50/20/30 (KEI2) and 40/30/30 (KEI3). The polymerization was performed in anhydrous 1,4-dioxane, preparing 4 wt% solution of NCAs. Then α-pinene and triphosgene were added. The reaction was carried out at 55 °C over 4 h. The product was precipitated with an excess of diethyl ether, then the precipitate was filtrated, washed with diethyl ether and then dried.

Molecular-weight characteristics (weight average and number average molecular weights, *M_w_* and *M_n_*, respectively, as well as dispersity, *Đ*) of polymers were evaluated by SEC. Shimadzu LC-20 Prominence system supplied with refractometric RID 10-A detector (Kyoto, Japan) and 7.5 mm × 300 mm Agilent PLgel MIXED-D column (Chrom Tech, Apple Valley, MN, USA) were applied for SEC analysis. DMF with 0.1 M LiBr was used as a mobile phase. The analysis was performed at 40 °C under 1.0 mL/min of the mobile phase flow rate. SEC LC Solutions software (Shimadzu, Kyoto, Japan) was used for calculations of *M_w_*, *M_n_* and *Đ* regarding to the calibration curve plotted for PMMA standards.

Additionally, the molecular weights and hydrodynamic radius *R*_h–D_ for macromolecules were measured by static and dynamic light scattering methods in solutions in DMSO at 21.0 °C. Light scattering was studied on a Photocor Complex unit (Photocor Instruments, Moscow, Russia); a Photocor-DL diode laser served as a light source (power of 5–30 mW, wavelength λ = 659.1 nm). The unit was calibrated by benzene (*R_V_* = 2.32 × 10^−5^ cm^−1^). The correlation function of the scattered light intensity was obtained with the use of a Photocor-PC2 correlator with 288 channels and was processed with DynalS software. In these solutions, the asymmetry of light scattering was absent; thus, *M_w_* of copolymers was determined by the Debay method. The refractive index increments were measured on a Refractometer RA-620 (KEM, Kyoto, Japan).

The Bzl- and Z-protective groups were removed by TFMSA/TFA mixture at a ratio 1/10 using known procedure [27]. After deprotection, the product was dissolved in DMF, placed into a dialysis bag (MWCO 1000), and dialyzed against water for 36 h.

The amino acid compositions of the polymers were determined using HPLC amino acid analysis after total hydrolysis of polypeptides up to free amino acids. The hydrolysis of 1 mg of a polypeptide dissolved in 1 mL of 6 M HCl with 0.0001% phenol was carried out in a sealed ampoule for 48 h at 110 °C. The solvent was evaporated several times with water to eliminate HCl and to reach neutral pH. The hydrolysates were analyzed using LC-20 Shimadzu system with refractometric RID-20A detector (all from Shimadzu, Kyoto, Japan) equipped with 4.6 × 125 mm Shodex IC YS-50 column, 5 µm beads (Showa Denko, Kyoto, Japan). The isocratic elution mode was applied and 3 mM H_3_PO_4_ solution was used as eluent. The mobile phase flow rate was 1.0 mL/min.

#### 2.2.2. Preparation and Characterization of Polypeptide Particles

Polymer nanoparticles were obtained by phase inversion during dialysis from DMSO to water followed by freeze drying for 2 days and finally dispersing for 30 s under sonication using 10% power of ultrasonic homogenizer (Bandelin Sonopuls HD2070, Berlin, Germany) at necessary concentration (0.20–1.50 mg/mL) in water or buffer of choice.

Average hydrodynamic diameter of particles (*D_H_*) and polydispersity index (PDI) were established at 25 °C by dynamic light scattering (DLS) Zetasizer Nano ZS equipped with a He–Ne laser beam at λ = 633 nm and a scattering angle of 173° (Malvern Instruments, Worcester, UK). DLS experiments were performed in deionized water, DMEM and PBS, pH 7.4, whereas the zeta-potentials were determined in deionized water. To study the pH effect, ζ-potential was measured in deonized water containing 10^−3^ M NaCl and adjusted with 0.1 M HCl/NaOH to pH 3–12.

The morphology was investigated by transmission electronic microscopy (TEM) using a JEM-2100 microscope (Jeol, Tokyo, Japan) operated at an acceleration voltage of 160 kV. The samples were prepared by dropping 3 μL of particles’ suspension (0.3 mg/mL) on copper grid covered with carbon and further staining with 3 *w*/*w*% uranyl acetate solution for 1 min. The grids were washed gently with pure water and dried for 30 min before the measurement.

Critical micelle concentration (CMC) was determined by conductometry. The measurements of conductivity were performed at 25 °C in the range of concentrations 0.5–35 µg/mL with the use of SevenCompact Cond meter S230 conductometer (Mettler Toledo, Columbus, OH, USA). CMC was determined as the intersection point of two linear sections of the conductivity vs. polymer concentration plots.

#### 2.2.3. Encapsulation and Release of RNA, Duplex Oligo-dT-dA and siRNA

Due to the presence of ε-amino groups of lysine in polypeptides obtained, negative oligo- and polynucleotides can easily bind to them due to the ionic interactions. Firstly, the suspension of polypeptide particles with concentration of 1 mg/mL was prepared and then diluted to a desired concentration. Then, the solution of oligonucleotide (0.1 nmol/µL) or siRNA (0.05 nmol/µL) was added quickly to suspension under stirring (Vortex, Thermo Fischer Scientific, Vantaa, Finland).

To determine the loading efficiency of polypeptide nanoparticles regarding to oligonucleotides we used Cy3-oligo-dT-dA. After encapsulation, non-bound Cy3-oligo-dT-dA was separated from nanoparticles by centrifugation (10,000× *g*) at 4 °C for 20 min in Amicon Ultra filter tubes with MWCO 30,000 (Merck). The filtrate containing free Cy3-oligo-dT-dA was collected and then analyzed using a Thermo Scientific Varioscan microplate reader at excitation and emission wavelengths of 550 and 570 nm, respectively. The amount of free Cy3-oligo-dT-dA was determined using a linear calibration plot and then the amount of loaded Cy3-oligo-dT-dA was calculated as a difference between initial and non-bound oligonucleotide amounts. The entrapment efficacy *(EE)* was calculated using the following equation:

(1) EE=(m1−m2)/m1  × 100%

where *m*1 is initial amount of Cy3-oligo-dT-dA, but *m*2 is the amount of non-bound Cy3-oligo-dT-dA.

In vitro release of Cy3-oligo-dT-dA from nanoparticles was investigated over 5 days. 100 µL of test formulation was diluted with 300 µL of the release medium (0.01 M Na-phosphate buffer, pH 7.4, cell culture medium DMEM or DMEM-F12 with 10% (*v*/*v*) fetal calf serum, FCS) and placed in Eppendorf filter tubes, which were incubated in a thermoshaker at 37 °C and stirring for 300 rpm.

After a certain period, the tubes were centrifuged at 10,000× *g* for 10 min. The filtrates that contained free Cy3-oligo-dT-dA were collected and fluorescence of Cy3 was analyzed using a fluorometer (λ_ex_ = 550 nm, λ_em_ = 570 nm). The amount of free Cy3-oligo-dT-dA was calculated using a linear calibration plot.

#### 2.2.4. Cytotoxicity of Particles

ARPE-19, HEK-293 and BEAS-2B were used for cytotoxicity evaluation. 10^4^ cells per well were cultured in a 96-well plate (200 μL/well) in DMEM-F12 containing 10% (*v*/*v*) fetal calf serum and 1% (*v*/*v*) penicillin/streptomycin (72 h, 37 °C, a humidified atmosphere of 5% CO_2_). After that, the cells were cultivated in the culture medium containing test empty polypeptide particles at the concentrations from 3 to 125 μg/mL for 24 and 72 h. The viability was evaluated using MTT-assay. For that, culture medium was aspirated and 100 μL/well of MTT solution (1.0 mg/mL in DMEM-F12) was added. The plate was incubated for 2 h. Finally, the solution was removed and 100 μL of DMSO was added to each well. After 10 min of gentle shaking, the solution absorbance was measured at 570 nm with the use of Fluoroscan Ascent microplate reader (Thermo Fisher Scientific Inc., Waltham, MA, USA). The relative cell viability (%) was calculated as following:

(2) Cell viability= (Asample – Ablanck)/ (Acontrol – Ablanck) × 100%

#### 2.2.5. Cellular Uptake

To visualize cell penetration of nanoparticles loaded with siRNA we used polypeptide particles loaded with Cy5 labeled VEGF siRNA. Approximately 10,000 ARPE-19 cells were seeded per well in a 96-well plate (Thermo Scientific^TM^ Nunc^TM^ MicroWell^TM^) in DMEM-F12. After 12 h, the medium was removed by aspiration and 90 µL serum-free DMEM-F12 medium with penicillin and streptomycin was added to each well. Peptide nanoparticles loaded with siRNA were added to cells (concentration of Cy-5 labeled siRNA was 1 nmol/mL). The cells were incubated with particles at 37 °C in serum-free medium for 4 h. Then the cells were washed with 1 M sodium chloride to remove remaining extra particles from cell surface, and the wells were filled with DMEM-F12 and the incubation continued for 44 h more. In each well the cells were fixed using 200 µL of 3.7% solution of formaldehyde in methanol. After that, the cells were washed three times with PBS.

We used Permeabilization Buffer (0.2% Triton X-100 in PBS) to permeabilize cell membranes. Cell nuclei were stained with Hoechst 33,258 (1 µg/mL) for 30 min according to the protocol published elsewhere [28]. After that, the cells were washed for three times with PBS and two times with water in order to remove crystals of salt.

The cell membranes were stained by CellMask Green Plasma Membrane Stain (Thermo Fischer Scientific, Paisley, UK), according to the manufacturer’s protocol. The study of cellular uptake was performed using CELENA S Digital Imaging System (Logos Biosystems, GE Healthcare, Anyang, Korea) and Cytell Cell Imaging instrument (GE Healthcare, Issaquah, WA, USA) by analysis of the fluorescence intensity of Cy5-siRNA.

To confirm particle penetration into the cells, we performed an additional experiment. For this purpose, we labeled polypeptide particles with Cy5 and used Cy3-oligo-dT-dA for encapsulation. 10 µL of 0.1 mg/mL solution of Cy5 in DMSO was added to 500 µL of 0.1 mg/mL suspension of nanoparticles in 0.01 M Na-phosphate buffer, pH 7.4, and stained for 1 h at 37 °C. Non-bound Cy5 was separated from nanoparticles by centrifugation (10,000× g) using filter tubes with MWCO 10,000 (Merck, Darmstadt, Germany) 7–8 times until the filtrate was free from Cy5. The encapsulation of Cy3-dT-dA and the cell experiments were the same as described above.

#### 2.2.6. VEGF Gene Silencing

The efficacy of siRNA, loaded in peptide nanoparticles, against VEGF was evaluated in ARPE-19 cells. Approximately 4 × 10^4^ ARPE-19 cells were seeded per well in a 24-well plate (Thermo Scientific^TM^ Nunc^TM^ MicroWell^TM^) in DMEM-F12/10% FBS (Biowest, Nuaille, France)/50 IU/mL penicillin/50 µg/mL streptomycin (BioloT) overnight. After that, the medium was removed by aspiration and 500 µL serum-free DMEM-F12 medium was added to each well. Then, 0.05 nmol of the siRNA loaded in 33.5 µg of polypeptide particles was added to each well (mass ratio particles:siRNA was equal to 4:1). The siRNA concentration was 100 nM and it was selected from data published elsewhere for ARPE-19 cells where the concentration range from 10 to 1000 nM was investigated [29,30]. The cells were incubated with particles at 37 °C for 4 h. Then the medium was replaced with fresh one and the incubation continued additionally for 44 h. To investigate RNA interference in the absence of VEGF we used the nanoparticles with scrambled siControl. The conditions were the same as for target siRNA.

#### 2.2.7. Total RNA Isolation, Reverse Transcription and Quantitative Real-Time PCR Analysis

The efficiency of VEGF silencing was estimated by measuring VEGF mRNA expression quantitatively using real-time reverse transcription polymerase chain reaction (RT-PCR). Glyceraldehyde-3-phosphate dehydrogenase (GAPDH) mRNA expression level was used as a housekeeping gene. After lysis of ARPE-19 cells, the total RNA was isolated using an RNA extraction kit from Biosilica (Novosibirsk, Russia) according to the manufacturer’s instructions and the RNA concentration was measured by the absorbance at 260 nm wavelength using an UV NanoDrop spectrometer (Thermo Scientific, Rockford, IL, USA). The ratio of absorbance at 260 nm and 280 nm for noncontaminated RNA samples was in the range of >1.8 and <2.2 [31]. The integrity of total RNA was determined by native 1% agarose gel electrophoresis [31]. Intact 28S and 18S rRNA were detected on a gel as sharp bands with intensity ratio ~2:1 and no signs of genomic DNA contaminations were observed. Then, 60 ng of cDNA was synthesized using the MMLV RT kit (Evrogen, Moscow, Russia). After that, 12 ng of cDNA was applied for RT-PCR with the respective VEGF forward, VEGF reverse, GADPH forward, and GADPH reverse primers. RT-PCR was performed using a qPCRmix-HS SYBR (Evrogen) according to the manufacturer’s instruction. The PCR performed for 40 amplification cycles of 95 °C for 30 s, 55 °C for 30 s, and 72 °C for 45 s. The amount of VEGF mRNA was determined relative to the amount of housekeeping gene glyceraldehyde 3-phosphate dehydrogenase (GAPDH) mRNA in the same sample by the equation X_0_/R_0_ = 2C_t_R−C_t_X, where X_0_ is the original amount of VEGF mRNA, R_0_ is the original amount of GAPDH mRNA, C_t_R is the C_t_ (cycle threshold) value for GAPDH, and C_t_X is the C_t_ value for VEGF.

#### 2.2.8. Western Blotting

To confirm the results of VEGF-silencing on protein level, western-blotting was performed as well. ARPE-19 cells were seeded on 6-well plate at a density of 8 × 10^5^ cells/well in DMEM-F12. After 12 h, the medium was aspirated, and 2 mL serum-free DMEM-F12 medium with penicillin and streptomycin was added. Then, 0.8 nmol of the siRNA loaded in polypeptide nanoparticles was added (mass ratio polymer:siRNA was 4:1). The nanoparticles with scrambled siRNA sequence were used as a control. For analysis of VEGF level suppression in a presence of nanoparticles loaded with siRNA, ARPE-19 cells were cultured for 5 days (instead of 2 days as for mRNA study) due to the slow turnover of the protein [13,32]. Afterwards, the cells were lysed with radioimmunoprecipitation assay (RIPA) buffer. Total protein in the lysate was quantified by Bradford assay using the albumin calibration curve as a standard. The equal amount of protein (20 μg) was separated on a 12% SDS-PAGE gel and electroblotted onto polyvinylidenedifluoride (PVDF) membrane (0.2 μm, Thermo Scientific, Rockford, IL, USA) at 400 mA for 20 min. The PVDF membrane was blocked with 5% (*w*/*v*) nonfat dried milk diluted in TBS buffer with 0.1% (*v*/*v*) Tween 20 for 1 h at room temperature. Recombinant humanized monoclonal anti-(human VEGF) antibody (1:20,000; Avastin, Roche, Welwyn Garden City, UK) and monoclonal mouse anti-(human β-actin) antibody (1:1000) (Santa Cruz Biotechnology, Heidelberg, Germany) were used as primary antibodies and horseradish peroxidase-linked rabbit anti-human IgG Fc antibody (1:10,000; Thermo Fisher Scientific) and rabbit anti-goat IgG (H + L) antibody (1:10,000; Thermo Fisher Scientific) were used as secondary antibodies. Blots were developed with enhanced chemiluminescence Amersham Hyperfilm ECL reagent and detection was done using Amersham Imager 600 Instrument (GE Healthcare, Little Chalfont, UK).

#### 2.2.9. Statistical Analysis

To analyze the statistical significance among the groups, one-way analysis of variants (ANOVA) in Excel with the XLSTAT was used. Data were expressed as mean ± SD (*n* = 3). *p* ≤ 0.05 was counted as a statistically significant.

## 3. Results and Discussion

### 3.1. Synthesis and Characterization of Polypeptides

Two series of polypeptides with different hydrophobic amino acid contents were synthesized by varying the initial ratio of amino acids in the synthesis. Polypeptides had a random primary structure that was generated during copolymerization of NCAs of l-Lys(Z), l-Glu(OBzl) and l-Phe/l-Ile via ROP technique [33]. Weight average molecular weight (*M_w_*), number average molecular weight (*M_n_*) and dispersity (*Ð*) of protected copolymers were evaluated using size-exclusion chromatography (SEC) in DMF with refractometric detection regarding poly(methyl methacrylate) standards (Table 1). Additionally, for some samples *M_w_* values were also determined by static light scattering (SLS) (Table 1 and Supplementary Materials Table S1) to testify the applicability of used SEC for such kind of polymers. *M_w_* values measured by both methods were found to be close. The polydispersity of the samples was in the range of 1.10–1.33 that corresponds to quite narrow molecular weight distribution.

The amphiphilic copolymers were prepared via deprotection of ε-amino groups of lysine and γ-carboxylic groups of glutamic acid. After deprotection, the copolymers acquired the tendency to self-assembly in aqueous media. The completeness of removal of the protective groups was verified by ^1^HNMR spectroscopy (Supplementary Materials Figure S1).

HPLC analysis of hydrolyzed polypeptide samples allowed for the determination of the ratio between amino acids (Table 2). The samples KEF1, KEF2, and KEI1-KEI3 were enriched with lysine whereas KEF3 was enriched with glutamic acid. Additionally, to evaluate polymer composition, the samples were analyzed by ^1^H-NMR spectroscopy. ^1^HNMR spectra and information on correlations of signals can be found in Supplementary Materials (Figures S2 and S3). For both Phe- and Ile-containing copolymers, the content of hydrophilic (Lys + Glu) and hydrophobic (Phe or Ile) amino acids (mol%) established by HPLC amino acid analysis was in an agreement with data obtained by ^1^HNMR spectroscopy. Due to the overlap of signals related to the carboxybenzyl and benzyl groups of Lys(Z) and Glu(OBzl), the separate calculation of Lys and Glu content could not be possible by ^1^HNMR method.

### 3.2. Preparation and Characterization of Polypeptide Particles

The preparation of nanoparticles (NPs) based on the synthesized polypeptides was carried out by gradient solvent inversion approach favoring to slow polymer self-assembly and followed by freeze-drying. Before application, the necessary amount of sample was redispersed in water or 0.01 M PBS via short-term ultrasonic exposure (30 s). Usually, the main force for the self-assembly of amphiphilic polymers is the interactions between hydrophobic regions of macromolecules in organic-aqueous or aqueous media to reduce the area of contact with water. At the same time, the hydrophilic fragments are exposed into aqueous medium because of their good solvation with water. In our amphiphilic macromolecules, except hydrophobic interactions, the electrostatic forces play a role due to the presence in polypeptides positively charged Lys and negatively charged Glu residues. Such complex interactions between polypeptide chains should provide high stability of self-assembled nanoparticles.

Size, surface charge and stability are among the most important parameters of nanoparticles, affecting cellular uptake and cytotoxicity. Various methods were used in this study to characterize the polypeptide nanoparticles obtained. Such parameters as hydrodynamic diameter (*D_H_*), polydispersity index (PDI) and electrokinetic potential (ζ-potential) of obtained nanoparticles were determined by dynamic light scattering (DLS) and electrophoretic light scattering, respectively (Table 3). The smallest particles with the average hydrodynamic diameter about 200 nm were formed when Lys/Glu ratio ranged from 1.9 to 2.8 (samples KEF1, KEI1 and KEI2). The same samples were also characterized with the lowest PDI. As expected from the copolymer’s composition, all samples, except KEF3, had positive ζ-potential, while KEF3 sample, enriched with glutamic acid (Table 2), was charged negatively. We chose the samples KEF1, KEI1 and KEI2 for further studies.

The critical micelle concentration (CMC) were determined for the selected polymers by conductometry method [34]. With increasing polymer concentration, the formation of thermodynamically stable aggregates starts and it is can be detected as a change in solution conductivity. The CMC values were calculated by a linear fitting of the two data subsets and calculating the concentration at their intersection (Supplementary Materials Figure S4). CMC values for KEF1, KEI1 and KEI2 were found to be close to each other and equal to 8.9, 10.6 and 6.2 mg/L, respectively. The values determined were close [35] or even lower [36] than these established for other amphiphilic random copolymers.

**Figure 1:**
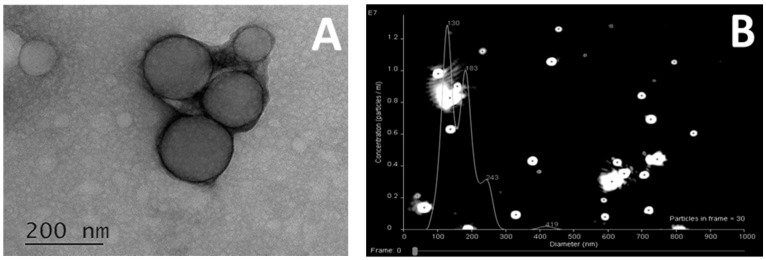
Size and structure of polypeptide nanoparticles (sample KEI2), investigated by TEM (**A**) and NTA (**B**).

Polypeptide nanoparticles were studied with nanoparticle tracking analysis (NTA) and transmission electron microscopy (TEM) to receive more information about their size, shape and structure. As an example, Figure 1 illustrates TEM and NTA images obtained for sample KEI2. According to TEM investigations, the nanoparticles of chosen sample represented spheres with a mean diameter of about 160 ± 50 nm. NTA allows for the determination of hydrodynamic diameter and size distribution profile of small particles that are hard to capture by DLS. According to NTA, there were three modes of nanoparticles with *D_H_* around 130, 180 and 240 nm. In turn, the DLS measurements gave the average value for the same sample equal to 180 nm with no detection of particles of different size (Supplementary Materials Figure S5). Similar results were obtained for two other selected samples (KEF1 and KEI1).

**Figure 2:**
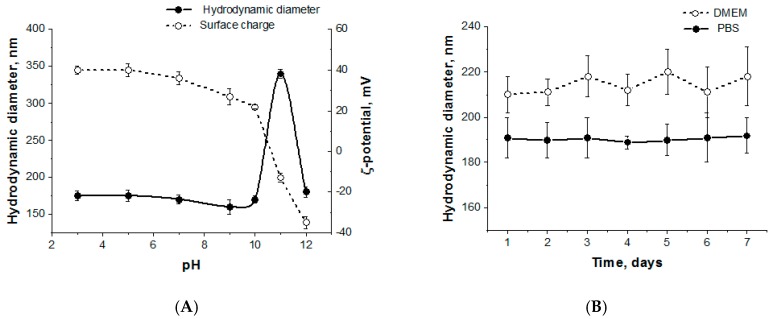
pH sensitivity (**A**) and stability (**B**) of polypeptide nanoparticles (sample KEI2).

The pH of solutions did not affect the particle size until it reached 10.5 (Figure 2A). At this point, ε-amino groups of lysine are deprotonated and became neutrally charged. As a result, an aggregation takes place. Such behavior was observed previously for random copolymers of lysine and phenylalanine [37]. In that case, the 10-fold increase in *D_H_* was detected. For terpolymers containing two oppositely charged amino acids (lysine and glutamic acid), the hydrodynamic diameter of the particles also increased but not so drastic: Only two-fold growth of *D_H_* was registered. Moreover, further pH elevation to 11 and 12 followed with charge switching to negative, −17 and −36 mV, respectively. The negative charge is explained by deprotonating of γ-carboxylic groups of glutamic acid, while amino group of lysine was uncharged. Moreover, the hydrodynamic diameter of nanoparticles at pH 12 returned to the initial value.

The stability of nanoparticles was studied in PBS and DMEM over 7 days. All samples proved to be colloidally stable, keeping their size and surface charge constant (Figure 2B).

### 3.3. Entrapment and Release of Duplex Oligo-dT-dA

**Figure 3:**
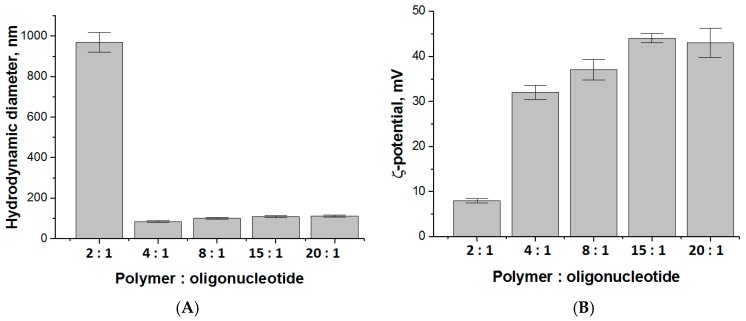
Hydrodynamic diameter (**A**) and surface charge (**B**) of polypeptide nanoparticles (sample KEI2), loaded with duplex oligo-dT-dA.

Entrapment efficacy and release kinetics are crucial characteristics for developed delivery system. At first, it was important to understand how the loading of oligonucleotide influences the physico-chemical characteristics of nanoparticles. For this purpose, duplex oligo-dT-dA was used as the cheaper and stable model of siRNA. The positively charged nanoparticles (sample KEI2) were loaded with negatively charged oligo-dT-dA due to polyelectrolyte interactions. The hydrodynamic diameter of the loaded particles as well as the surface charge depended on polymer:oligonucleotide ratio, while mean PDI was constant and equal to 0.18±0.01. The particle’s hydrodynamic diameter decreased gradually and the surface charge lowered steadily with the increase of encapsulated duplex oligo-dT-dA (Figure 3). A significant aggregation was observed when the polymer:oligonucleotide ratio reached 2:1 and the surface charge dropped to +8 mV. Thus, it can be deduced that polymer:oligonucleotide ratio 4:1 provided the smallest particles with a noticeable surface charge.

**Figure 4:**
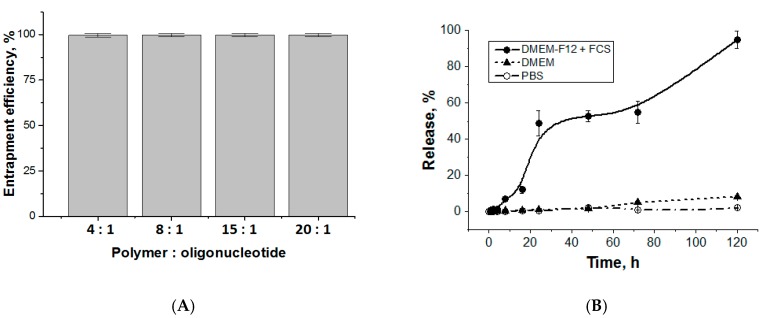
Entrapment efficacy (**A**) and release (**B**) of duplex oligo-dT-dA (sample KEI2) from the complex (polymer:oligo-dT-dA ratio equal to 4:1).

To compare the entrapment efficacy and to study the oligonucleotide release kinetics, we used the same oligonucleotide but labeled with Cy3 fluorescent dye. Since the particle size was stable until the polymer:oligo-dT-dA ratio reached 2:1, the oligonucleotide encapsulation was carried out in the range of this ratio from 4:1 to 20:1. Since pH does not affect particle size and morphology (except at pH 10.5) the encapsulation was performed in pure water. Almost 100% of labeled oligonucleotide was entrapped into polypeptide nanoparticles, regardless polymer:oligonucleotide ratio (Figure 4A). Therefore, the ratio 4:1 was appeared to be suitable for further experiments.

The release of duplex oligo-dT-dA was studied for the system obtained at polymer:oligo-dT-dA ratio = 4:1. A simple buffer system (PBS, pH 7.4), a complex medium containing amino acids, vitamins and inorganic salts (DMEM) and DMEM-F12 containing 10% of fetal calf serum (DMEM-F12 + FCS) were used as the media for experiments. About 50% of oligonucleotide duplex was released over 24 h in DMEM-F12 + FCS (Figure 4B). After 5 days of incubation in DMEM-F12 + FCS at 37 °C, the release reached 95%. However, oligonucleotide duplex did not escape polymer particles in PBS during 5 days that means good storage stability of formulation. At the same time, the release in DMEM without serum was negligible. The release in DMEM-F12 + FCS medium is explained by the presence of different negatively charged components like proteins and vitamins at relatively high concentrations that could interact with polypeptide and, therefore, gradually replace oligonucleotide. Since there are a lot of proteins and other molecules with negative charge in the cytoplasm, we suppose that this mechanism of substitution will work also inside a cell to release siRNA.

Additionally, we prepared the polyplex of poly-L-lysine and duplex oligo-dT-dA in the same ratio (4:1) and studied the release of oligonucleotide in DMEM and DMEM-F12 + FCS over 5 days (37 °C). It was established that the oligo-dT-dA release in DMEM did not exceed 2.5%. The presence of FCS in medium slightly facilitated the release of target compound, however, even in this case it did not exceed 8% (Supplementary Materials Figure S6). Thus, the combination of positively and negatively charged amino acids, e.g., lysine and glutamic acid, indeed facilitate the release of oligonucleotides in comparison with poly-L-lysine.

### 3.4. Cytotoxicity

**Figure 5:**
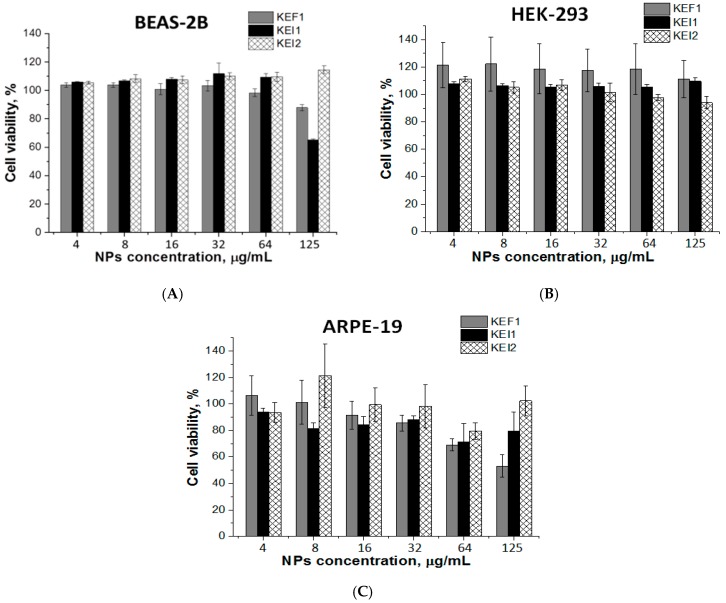
Cytotoxicity of the nanoparticles in BEAS-2B (**A**), HEK-293 (**B**) and ARPE-19 (**C**) cells (72 h).

Since the stability of nanoparticles for 7 days in the test medium was proven with DLS, we proceeded the investigation of their cytotoxicity in cultured cells (Figure 5 and Supplementary Materials Figure S7). For this purpose, MTT assay was performed using three cell lines, e.g., HEK-293, BEAS-2B and ARPE-19 cells. The cells were incubated in the presence of polypeptide nanoparticles (KEF1, KEI1, KEI2) for 72 h. It was established that the samples KEF1 and KEI2 were not toxic for HEK-293 and BEAS-2B cells in the concentration range of 4–125 μg/mL. For the sample KEI1, modest cytotoxicity was observed at concentration 125 μg/mL (viability 65 ± 1%). In the case of ARPE-19, cell viability was higher than 75% for KEI1 and KEI2 samples in the whole tested concentration range. The sample KEF1 demonstrated evident cytotoxicity at the concentration of 125 μg/mL.

### 3.5. VEGF Gene Silencing

To investigate VEGF gene silencing, KEF1, KEI1 and KEI2 nanoparticles were selected as the delivery systems for anti-VEGF siRNA. We used ARPE-19 cells that express VEGF constitutively. The experiment was carried out using the polymer:siRNA mass ratio of 4:1; the concentration of polymer particles used for experiment was 67 µg/mL. RNA interference of anti-VEGF siRNA was analyzed by RT-PCR.

**Figure 6:**
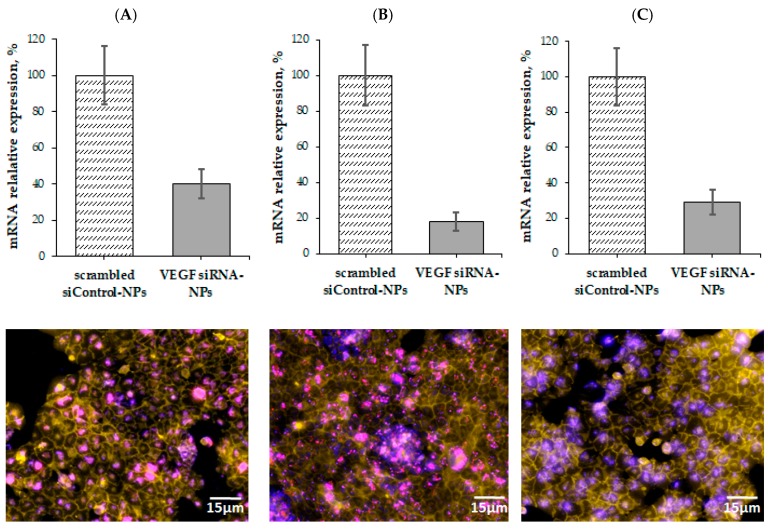
mRNA interference by anti-VEGF siRNA delivered by KEF1 (**A**), KEI1 (**B**) and KEI2 (**C**) nanoparticles (polymer:siRNA ratio = 4:1) in the ARPE-19 cells (mean ± SD, *n* = 3). Cell membranes were stained with Cell Mask Green Plasma Membrane dye (green-yellow color), cell nuclei were stained with Hoechst 33,258 (blue color). Cy3 labelled siRNA was depicted in pink color.

All investigated samples demonstrated strong inhibitory effect on mRNA expression (Figure 6). The minimal inhibitory effect of 60% was reached, using KEF1 as a delivery system, while KEI1 and KEI2 inhibited VEGF gene expression more effectively (*p* < 0.01) (Figure 6). In particular, VEGF silencing reached with the use of KEI1 and KEI2 siRNA delivery systems corresponded to 82 and 71%. For comparison, 40–70% GFP gene knockdown was reported by Zhu et al. for the application of poly(dimethylaminoethyl methacrylate)-b-polycaprolactone-b-poly(dimethylaminoethyl methacrylate) systems for delivery of siRNA [38]. Wei et al. reported the application of chitosan-based systems for delivery of mTERT siRNA. The reached gene silencing was equal to 56% [39]. As to VEGF knockdown, the delivery systems based on poly(ε-caprolactone)- polyethyleneglycol-poly(l-histidine) provided up to 55% VEGF gene silencing in MCF-7 and HUVEC cells (siRNA concentration and incubation time with cells were 100 nM and 5 days, respectively) [13]. Thus, the polypeptide systems, developed in present work, can be considered as efficient for intracellular siRNA delivery and VEGF gene knockdown.

Fluorescence microscopy images of ARPE-19 cells treated with Cy5-siRNA–polypeptide particles showed effective cell penetration of Cy5-labeled siRNA. Most siRNAs were located in the cytoplasm (Figure 6). According to the fluorescent images, the delivery of Cy5-labled siRNA with KEI1 nanoparticles followed with much more pronounced aggregation of NPs. Taking this into account as well as gene silencing results and the data on cytotoxicity, KEI2 sample was selected for further western blotting analysis.

**Figure 7:**
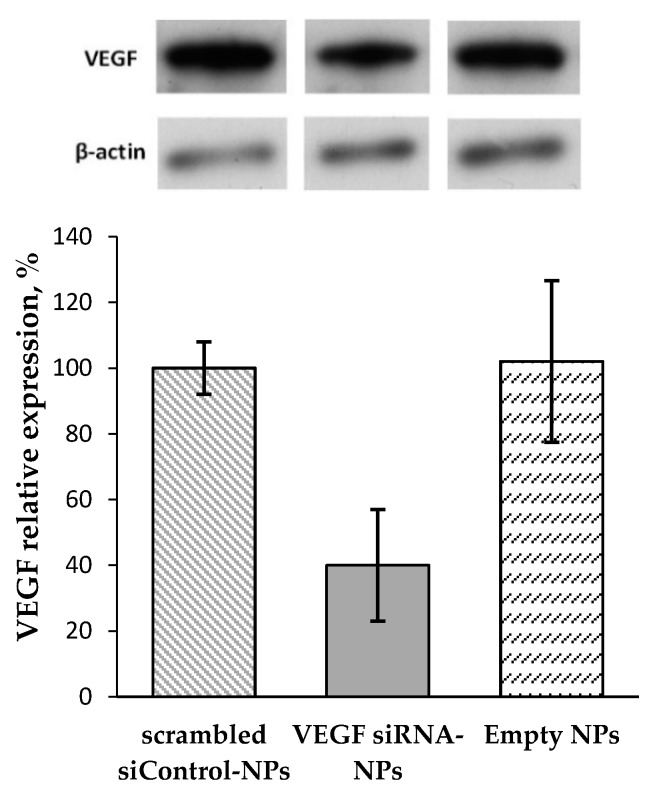
VEGF protein expression in the ARPE-19 cells determined by western blotting (KEI2 NPs).

To verify the suppression of VEGF expression due to the gene silencing the western blotting was carried out. For this, KEI2 nanoparticles loaded with siRNA was used as sample whereas scrambled siRNA loaded in the same kind of NPs was applied as negative control. Additionally, empty NPs were also applied as control to reveal if they can influence on the process under study. According to the results obtained, the expression of VEGF under siRNA delivery system application was suppressed approximately by 60% (*p* < 0.03) (Figure 7). The results obtained are in a good agreement with data on RNA interference evaluated by RT-PCR where the same sample provided gene silencing by 70%. No suppressing effect was detected for both empty NPs and scrambled siControl-NPs.

## 4. Conclusions

In this study, polymers based on lysine, glutamic acid and phenylalanine/isoleucine were developed for the delivery of oligonucleotides. The polymers self-assembled into spherical particles with average hydrodynamic diameters of 180–900 nm that were colloidally stable in PBS and cell culture medium (DMEM). The model duplex of oligonucleotides (oligo-dT-dA) was encapsulated in the polymer nanoparticles up to 4:1 mass ratio (polymer:oligo-dT-dA) without any size change in nanoparticles. The interaction between oligonucleotide duplex and the polymer was strong and no oligonucleotide release in PBS was detected, but the presence of various negatively charged components like proteins, vitamins and amino acids in DMEM-F12 + FCS enhanced the release and 56% of oligonucleotide was freed over 48 h. The cell viability test demonstrated the absence of cytotoxicity for the sample KEI2 for three cell lines whereas for some other samples slight toxic effect was detected. The efficient VEGF gene silencing was established at the application of developed polypeptide nanoparticles as siRNA delivery systems.

## Figures and Tables

**Table 1:** Molecular-weight characteristics of protected polypeptides determined by SEC and SLS.

Sample	Polymer Characteristics			
	SEC			SLS
	*M_n_*	*M_w_*	*Ð*	*M_w_*
*P(Lys(Z)_n_-co-Glu(OBzl)_m_-co-Phe_k_)*				
KEF1	20,170	23,600	1.17	23,000
KEF2	9400	17,000	1.80	17,500
KEF3	10,000	13,200	1.32	17,400
*P(Lys(Z)_n_-co-Glu(OBzl)_m_-co-Ile_k_)*				
KEI1	21,350	28,400	1.33	18,800
KEI2	16,090	21,240	1.32	17,100
KEI3	17,560	22,300	1.27	-

**Table 2:** Composition of amphiphilic amino acid polypeptides.

Sample	Determined Polymer Composition (mol%)				
	HPLC			^1^HNMR	
	*Lys*	*Glu*	*Phe*/*Ile*	*Lys* + *Glu*	*Phe*/*Ile*
*P(Lys_n_-co-Glu_m_-co-Phe_k_)*					
KEF1	55	25	20	76	24
KEF2	75	9	16	75	25
KEF3	21	54	25	64	36
*P(Lys_n_-co-Glu_m_-co-Ile_k_)*					
KEI1	66	16	18	85	15
KEI2	57	31	12	88	12
KEI3	54	36	10	-	-

**Table 3:** Characteristics of polypeptide nanoparticles obtained (DLS, in 0.01 PBS, pH 7.4).

Sample	Particle Characteristics		
	*D_H_*, nm	PDI	ζ-Potential, mV
*P(Lys_n_-co-Glu_m_-co-Phe_k_)*			
KEF1	200 ± 8	0.22	+12 ± 2
KEF2	550 ± 14	0.31	+18 ± 5
KEF3	900 ± 38	0.39	−6 ± 3
*P(Lys_n_-co-Glu_m_-co-Ile_k_)*			
KEI1	232 ± 11	0.14	+49 ± 2
KEI2	180 ± 19	0.19	+45 ± 3
KEI3	440 ± 31	0.30	+31 ± 5

## Supplementary Materials

The following are available online at https://www.mdpi.com/1999-4923/12/1/39/s1, Figure S1: ^1^HNMR spectrum of deprotected sample KEI1, Figure S2: ^1^HNMR spectrum of protected sample KEF1, Figure S3: ^1^HNMR spectrum of protected sample KEI2, Figure S4: Dependences of conductivity on polymer concentration: (a) sample KEF1 and (b) sample KEI1, Figure S5: DLS analysis of polymer nanoparticles prepared from P(Lys-co-Glu-co-Ile), sample KEI2, Figure S6: Release of duplex oligo-dT-dA from the complex with poly-l-lysine:oligonucleotide (ratio 4:1), Figure S7: Nanoparticle’s cytotoxicity in BEAS-2B and HEK-293 (a–c) and ARPE-19 cells (d) (24 h): (a) sample KEF1, (b) sample KEI1, (c) sample KEI2 and (d) sample KEI2, Table S1: Data of static (SLS) and dynamic (DLS) light scattering of polymer solutions (DMSO).
