# Supplementary material for: Amphiphilic Polypeptides for VEGF siRNA Delivery into Retinal Epithelial Cells
Source: Pharmaceutics. 2020 Jan 2;12(1):39. doi: 10.3390/pharmaceutics12010039 (PMC7022581; doi:10.3390/pharmaceutics12010039)
Supplement: Supplementary file 1 [file pharmaceutics-12-00039-s001.pdf]

pharmaceutics

Supplementary Materials: Amphiphilic Polypeptides for VEGF siRNA Delivery into Retinal Epithelial Cells

Olga Osipova, Vladimir Sharoyko, Natalia Zashikhina, Natalya Zakharova, Tatiana Tennikova, Arto Urtti and Evgenia Korzhikova-Vlakh

1. Polymer Characterization

1.1. Static and Dynamic Light Scattering of Polymer Solutions

Table S1. Data of static (SLS) and dynamic (DLS) light scattering of polymer solutions (DMSO).

Sample *	SLS			DLS
	dn/dc, cm³/g	M_w	A_2, cm³·mol·g⁻²	$R_{h\text{-}D}$, nm
KEF1	0.0542	23000	−5.45×10⁻³	2.2
KEF2	0.0513	17500	−2.04×10⁻³	2.2
KEF3	0.0574	17400	−1.75×10⁻³	2.2
KEI1	0.0537	18800	−2.69×10⁻³	1.0
KEI2	0.0608	17100	−2.13×10⁻³	1.4

* Polymers used in protected forms as P(Lys(Z)-*co*-Glu(OBzl)-*co*-Phe) and P(Lys(Z)-*co*-Glu(OBzl)-*co*-Ile).

1.2. ¹H NMR Spectroscopy

Poly(L-lysine-co-L-glutamic acid-co-L-isoleucine)

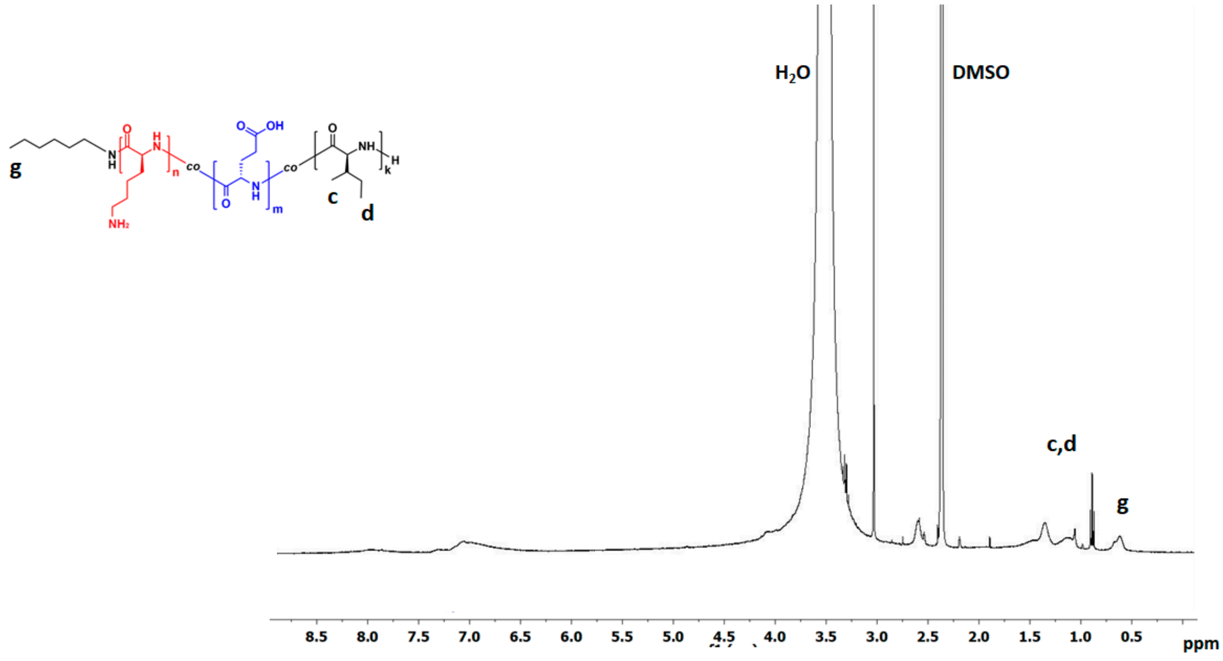

Figure S1. ¹HNMR spectrum of deprotected sample KEI1.

Poly(L-ε-carboxybenzyl-lysine-co-L-γ-benzyl-glutamic acid-co-L-phenylalanine)

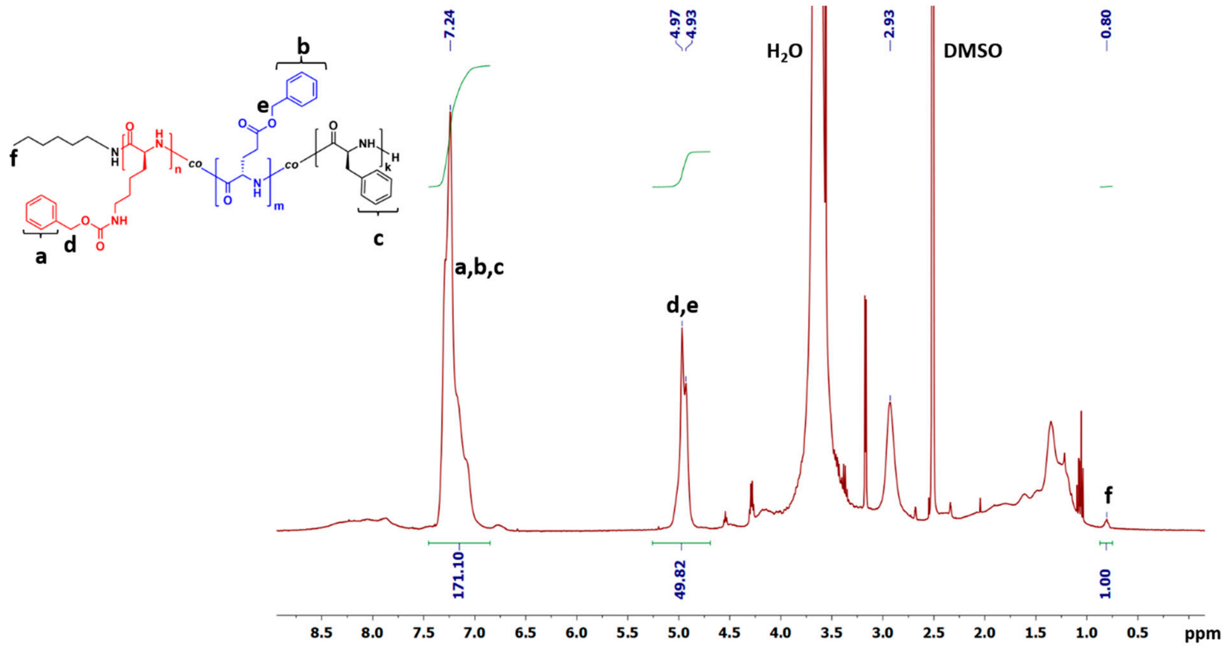

Figure S2. ^{1}HNMR spectrum of protected sample KEF1.

Polymer composition calculation was carried out using following equations:

$$[\boldsymbol{Phe}],\% = \left(\frac{(I(Lys(Z))_{6.9-7.4\text{ ppm}} + I(Glu(OBzl))_{6.9-7.4\text{ ppm}} + I(P\square e)_{6.9-7.4\text{ ppm}})/5 - ((I(Lys(Z))_{4.7-5.2\text{ ppm}} + I(Glu(OBzl))_{4.7-5.2\text{ ppm}})/2}{(I(Lys(Z))_{6.9-7.4\text{ ppm}} + I(Glu(OBzl))_{6.9-7.4\text{ ppm}} + I(P\square e)_{6.9-7.4\text{ ppm}})/5}\right) \times 100\% \quad (\mathbf{1}),$$

where [*Phe*], % - molar fraction of *Phe* in the copolymer, $I(Lys(Z))_{6.9\text{-}7.4\text{ ppm}}$, $I(Glu(OBzl))_{6.9\text{-}7.4\text{ ppm}}$ and $I(Phe)_{6.9\text{-}7.4\text{ ppm.}}$ are relative integral areas of 5 aromatic protons of *Phe*, *Z*- and *OBzl*-groups of polymer at 6.9–7.4 ppm, $I(Lys(Z))_{4.7\text{-}5.2\text{ ppm}}$ and $I(Glu(OBzl))_{4.7\text{-}5.2\text{ ppm}}$ are relative integral areas of 2 CH_2 protons of *Z*- and *OBzl*-groups of polymer at 4.7–5.2 ppm.

Poly(L-ε-carboxybenzyl-lysine-co-L-γ-benzyl-glutamic acid-co-L-isoleucine)

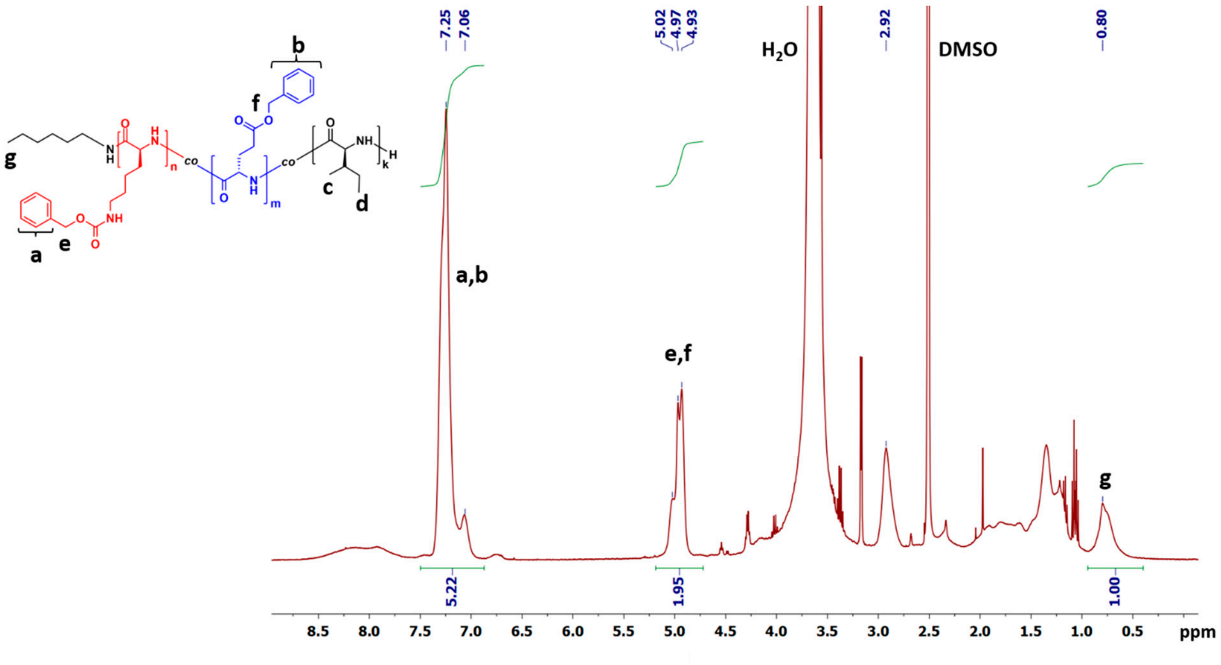

Figure S3. ^{1}HNMR spectrum of protected sample KEI2.

Polymer composition calculation was carried out using following equations:

$$[Ile],\% = \left(\frac{(I(Ile)_{0.5-0.9\ \text{ppm}})/6}{\left(I\left(Lys(Z)\right)_{6.9-7.5\ \text{ppm}} + \left(I\left(Glu(OBzl)\right)_{6.9-7.5\ \text{ppm}}/5\right)\right.}\right) * 100\% \quad (5),$$

Where [*Ile*], % - molar fraction of *Ile* in the copolymer, $I(Ile)_{0.5\text{-}0.9\ \text{ppm}}$ is relative integral area of 6 CH_3 protons of *Ile* at 0.5–0.9 ppm and $I(Lys(Z))_{6.9\text{-}7.5\ \text{ppm}}$ and $I(Glu(OBzl))_{6.9\text{-}7.5\ \text{ppm}}$.are relative integral areas of 5 aromatic protons of *Z*- and *OBzl*-groups of polymer at 6.9–7.5 ppm.

2. Determination of Critical Micelle Concentration

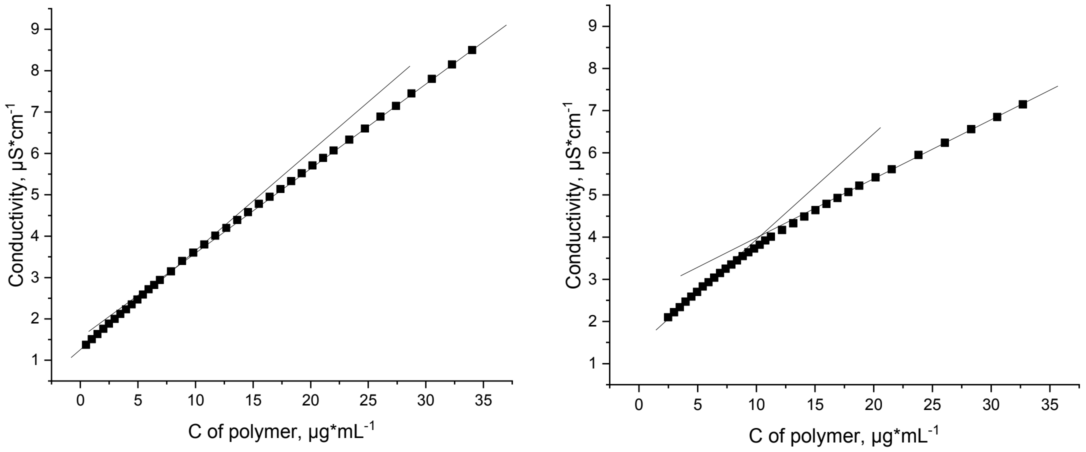

(**a**) (**b**)

Figure S4. Dependences of conductivity on polymer concentration: (**a**) sample KEF1 and (**b**) sample KEI1.

3. Dynamic Light Scattering

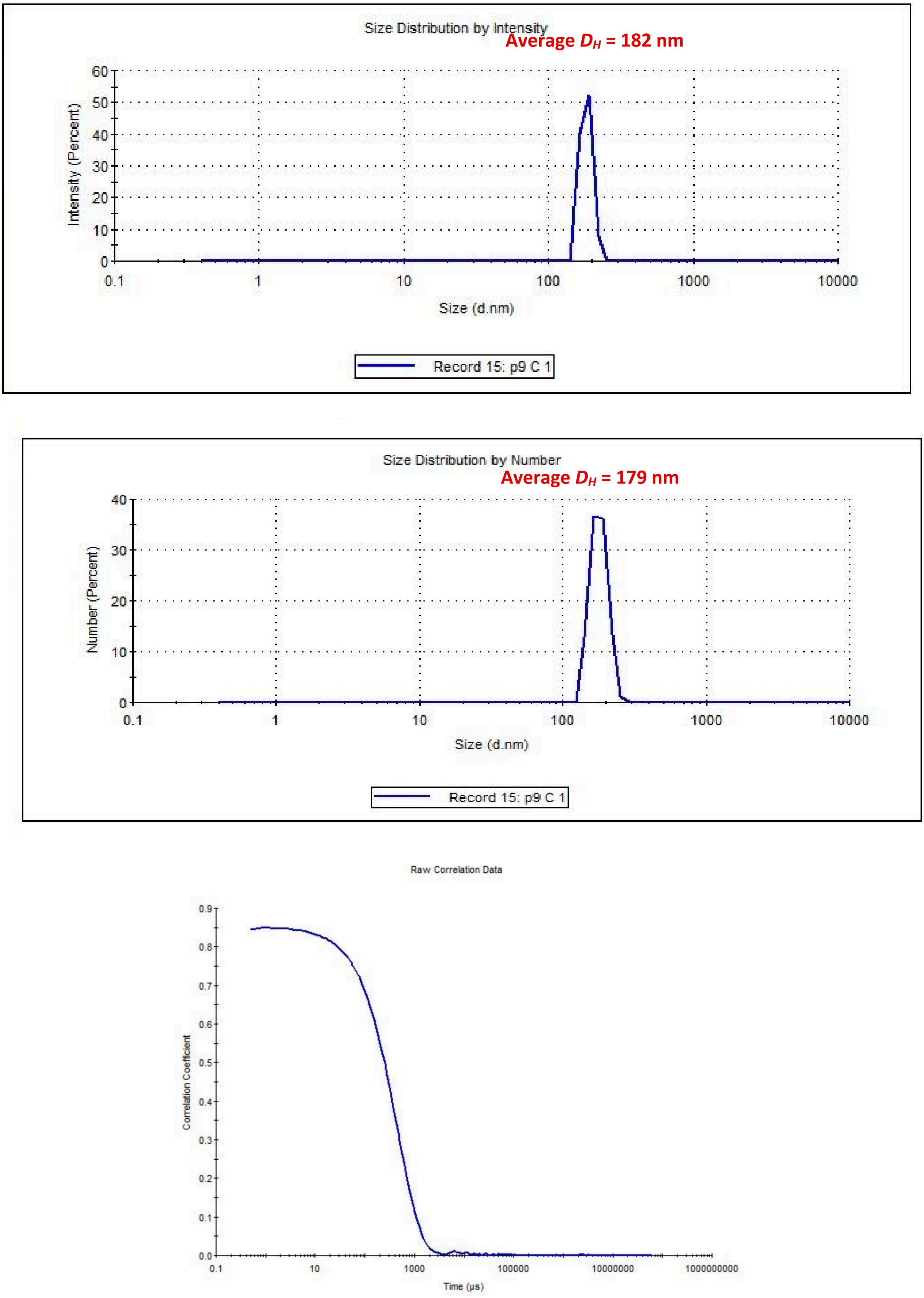

Figure S5. DLS analysis of polymer nanoparticles prepared from P(Lys-co-Glu-co-Ile), sample KEI2.

4. Release of duplex oligo-dT-dA from polyplex with poly-L-lysine

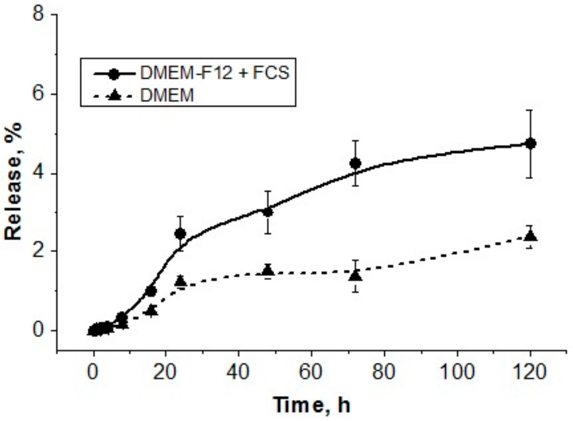

Figure S6. Release of duplex oligo-dT-dA from the complex with poly-L-lysine:oligonucleotide (ratio 4:1).

5. Cytotoxicity of Nanoparticles

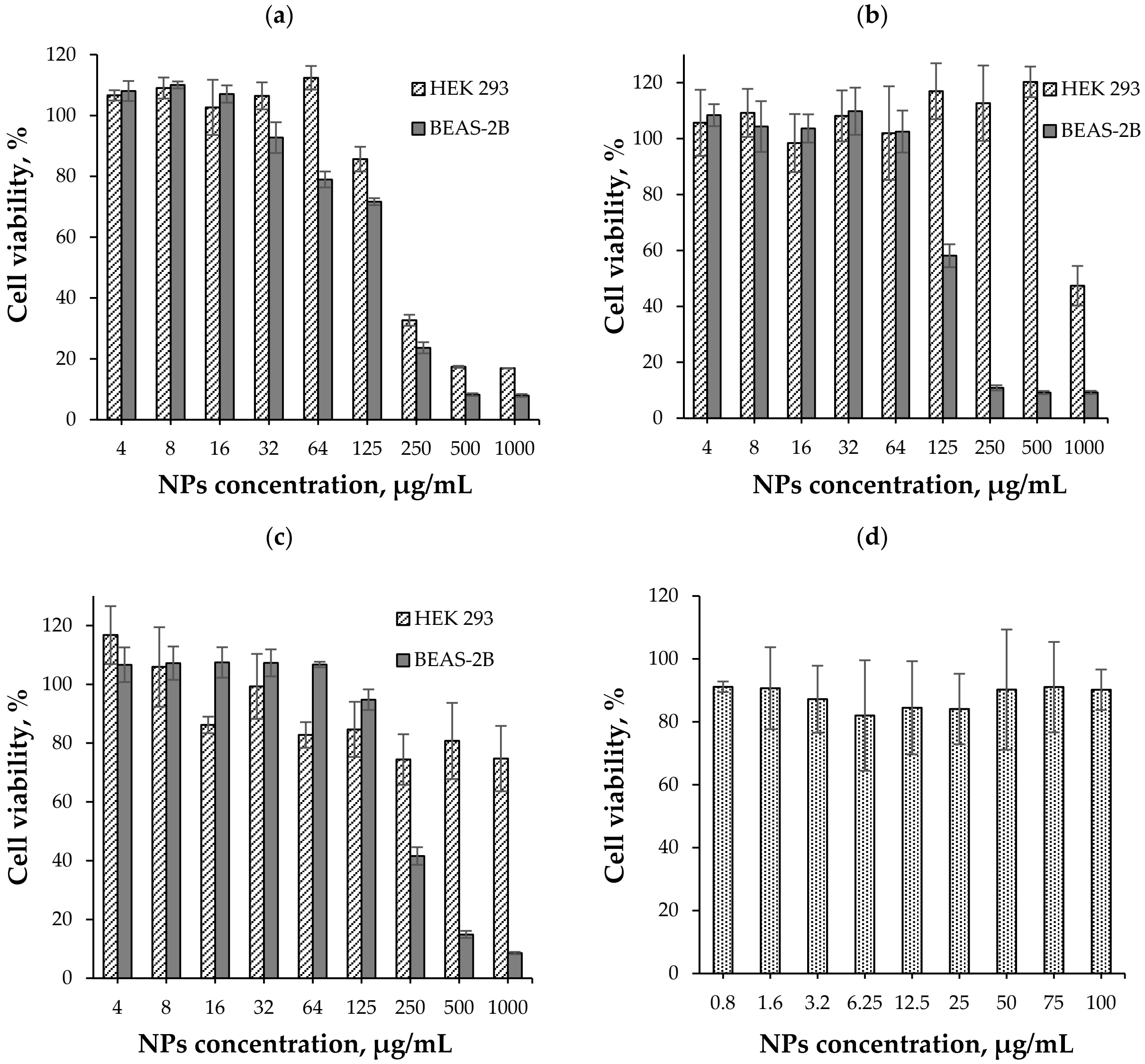

Figure S7. Nanoparticle's cytotoxicity in BEAS-2B and HEK-293 (**a**–**c**) and ARPE-19 cells (**d**) (24 h): (**a**) sample KEF1, (**b**) sample KEI1, (**c**) sample KEI2 and (**d**) sample KEI2.
